# Research Progress of Group II Intron Splicing Factors in Land Plant Mitochondria

**DOI:** 10.3390/genes15020176

**Published:** 2024-01-28

**Authors:** Xiulan Li, Yueshui Jiang

**Affiliations:** School of Life Sciences, Qufu Normal University, Qufu 273165, China; lxljewel@163.com

**Keywords:** group II intron, plant mitochondria, RNA splicing, protein splicing factor, RNA structure

## Abstract

Mitochondria are important organelles that provide energy for the life of cells. Group II introns are usually found in the mitochondrial genes of land plants. Correct splicing of group II introns is critical to mitochondrial gene expression, mitochondrial biological function, and plant growth and development. Ancestral group II introns are self-splicing ribozymes that can catalyze their own removal from pre-RNAs, while group II introns in land plant mitochondria went through degenerations in RNA structures, and thus they lost the ability to self-splice. Instead, splicing of these introns in the mitochondria of land plants is promoted by nuclear- and mitochondrial-encoded proteins. Many proteins involved in mitochondrial group II intron splicing have been characterized in land plants to date. Here, we present a summary of research progress on mitochondrial group II intron splicing in land plants, with a major focus on protein splicing factors and their probable functions on the splicing of mitochondrial group II introns.

## 1. Introduction

Mitochondria are proposed to originate from α-proteobacteria through endosymbiosis [1]. During endosymbiosis and evolution, most ancestral bacterial genes have been either lost or transferred into the host nuclear genomes, leading to nearly all mitochondrial proteins being encoded by the nuclear genes. As a result of proteobacterial origin, and deletion/insertion of sequences or horizontal gene transfer during long-term evolution, introns are commonly present in the mitochondrial genomes of land plants. According to their RNA-folding patterns and splicing mechanisms, introns in plant mitochondria are classified into two groups, I and II. Most mitochondrial introns in land plants are group II introns, and only a single group I intron is present in the mitochondrial *cox1* of some flowering plants [2,3]. Correct excision of these introns is critical to the gene expression and biological function of mitochondria, and the growth and development of plants. Canonical group II introns are self-splicing introns that can remove themselves from pre-RNAs *in vivo*. However, group II introns in plant mitochondria have degenerated, resulting in the lack of regions required for their self-splicing and the loss of their ability to self-splice [4]. Instead, splicing of plant mitochondrial group II introns is promoted by numerous nuclear- and mitochondrial-encoded protein cofactors [5]. So far, some proteins from diverse families have been found with functions in mitochondrial group II intron splicing in land plants. Here, we summarize research progress on mitochondrial group II intron splicing in land plants, with a major focus on protein splicing factors and their probable functions in the splicing of mitochondrial group II introns.

## 2. Group II Introns

### 2.1. Group II Intron Structure

**Figure 1:**
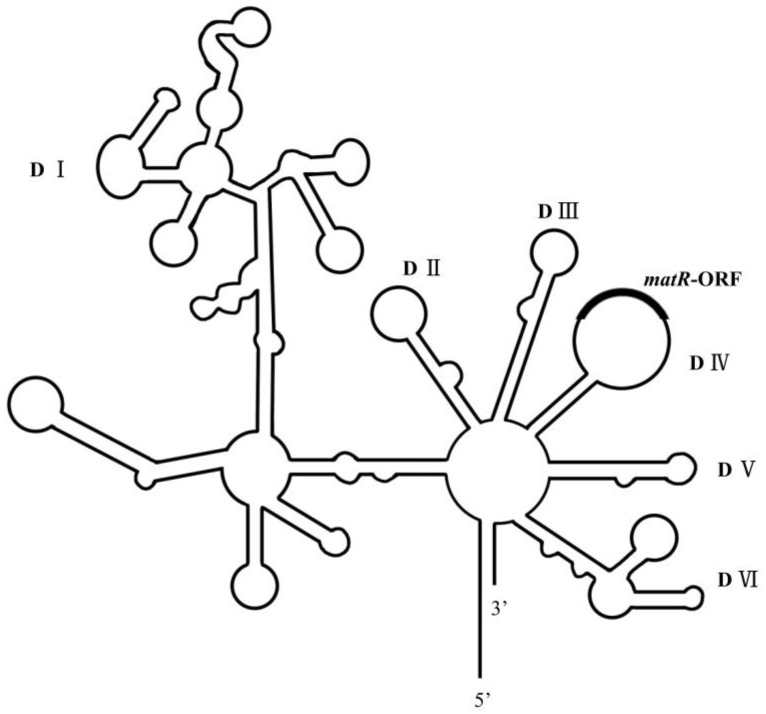
Diagrammatic sketch of *nad1* intron 4 predicted secondary structure. Six domains (DI–VI) of introns and the open reading frame (ORF) of *matR* are labeled.

Group II intron RNAs are characterized by a conserved secondary structure that consists of six stem-loop domains (DI to DVI) extending from a wheel [6] (Figure 1). DI is the largest domain, which interacts with other domains of group II introns and has functions in RNA folding [7]. DII and DIII are smaller domains, and they interact with each other to form key elements of the active site of group II introns [8]. DIV is the most diverse domain in different group II introns, and the ancestral DIV often carries a sequence expressing an intron-encoded protein (IEP). IEPs are multi-functional proteins with reverse transcriptase and maturase activities and are vital to the self-splicing of group II introns. DV is the most conserved domain, mostly composed of a 34 bp stem-loop structure. DV has a catalytic triad AGC and a binding site for Mg^2+^ ions, and it can interact with DI to form a catalytic core [9]. DVI harbors a branch-point adenosine that is generally located 7–8 bp upstream of the 3′ end of the intron. Not all group II introns in plant mitochondria possess conventional secondary structural features [2]. For example, *nad1* intron 1 has a larger DV loop and a DVI without a branch-point adenosine. *nad1* intron 2 has a short and strongly base-paired DVI. *nad4* intron 2 does not have a branch-point adenosine residue at the expected position.

### 2.2. Group II Intron Splicing

**Figure 2:**
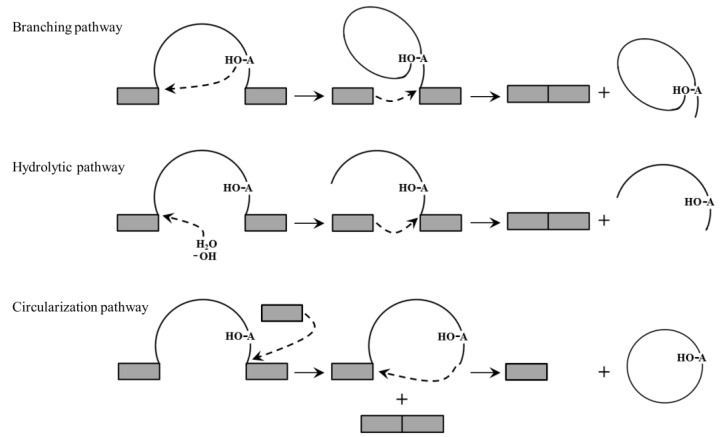
Three splicing pathways of group II introns. The grey boxes represent the exons. The curves represent the introns. The dashed arrows represent the transesterification reactions. The branch-point adenosine in DVI is labeled.

Group II introns are commonly spliced by the branching pathway containing the same two-step transesterification reactions as those of the spliceosomal introns in nuclei (Figure 2). Firstly, the 2′-OH of the branch-point adenosine attacks the phosphate at the 5′ splice site and attaches to the 5′ end of the intron, forming a free 3′-OH at the 5′ splice site and a lariat intermediate composed of an intron and a 3′ exon. Secondly, the free 3′-OH of the 5′ exon attacks the phosphate at the 3′ splice site, with the connection of the 5′ and 3′ exons and the releasing of the intron in a lariat form [10]. In the mitochondria of land plants, most group II introns are spliced by the branching pathway, and several of them are spliced through the hydrolytic pathway or the circularization pathway. For example, because of the lack of a branch-point adenosine in DVI, splicing of *nad1* intron 1 proceeds through the hydrolytic pathway [2]. During this pathway, H_2_O or -OH, instead of the branch-point adenosine in DVI, attacks the phosphate at the 5′ splice site and the intron is released in a linear form (Figure 2). Splicing of *nad1* intron 2 in wheat mitochondria is accomplished through the circularization pathway [6] In the first step, the free 3′-OH of an external free exon proposed to be generated by the spliced-exon reopening reaction attacks the phosphate at the 3′ splice site, generating ligated exons and a splicing intermediate. In the second step, the 2′-OH at the 3′ end of the intron attacks the phosphate at the 5′ splice site, releasing an independent exon and an intron in a circular form (Figure 2).

*In vivo*, the self-splicing and mobility of group II introns need the aid of IEPs. IEPs are multi-functional proteins and usually have a reverse transcriptase (RT) domain and an RNA-binding (X) domain at the N-terminus. The RT domain participates in the transcription of intron RNAs into DNAs. The X domain is related to RNA splicing and maturase activity. Some IEPs also contain a DNA-binding (D) domain and an endonuclease (En) domain following the X domain, both of which are critical for the mobility of group II introns. During the self-splicing and retrotransposition of group II introns, IEP functions as maturase and reverse transcriptase, recognizing its parent intron RNA and forming an IEP–intron ribonucleoprotein complex to promote splicing and reverse splicing of group II introns [11]. For group II introns in plant mitochondria, the coding sequences of IEPs have been lost or degenerated, resulting in the lack of their ability to self-splice. Instead, the splicing of group II introns in plant mitochondria is promoted by proteins encoded by nuclear and mitochondrial genes [5].

## 3. Splicing Factors of Mitochondrial Group II Introns in Land Plants

### 3.1. Maturase

In the mitochondrial genomes of angiosperms, only one *maturase-related* (*matR*) gene has been maintained within intron 4 of *nad1* [12,13]. MatR encoded by the *matR* gene is well conserved in angiosperms; it contains a shortened RT domain, an intact X domain, and fragments of the D/En motif [5,14]. MatR in angiosperms is closely associated with maturase encoded by bacterial group II introns [15], thus it is proposed to have similar functions in angiosperms. In brassicaceae, MatR was found to be related to the splicing of many group II introns in mitochondria, and its host *nad1* intron 4 is also included [14].

In addition to *matR* in mitochondrial genomes and *matK* in chloroplast genomes, there are four maturase genes designated *nMat* 1 to 4 in the nuclear genomes of angiosperms. *nMat* genes exist as standalone open reading frames and encode proteins closely associated with the maturases encoded by group II introns [16,17,18]. Based on their topology and proposed evolutionary origins, four nMATs encoded by *nMat* genes are divided into type I and type II maturases [16,17]. nMAT1 and nMAT2 are type I maturases, which contain the RT domain and the X domain but have lost the D/En motif, whereas nMAT3 and nMAT4 are type II maturases, harboring the RT domain, the X domain, and a predicted non-functional D/En motif. It is thus expected that all four nMATs in angiosperms have kept splicing activities but lack mobility-associated functions. Subcellular localization experiments indicate that nMAT 1 to 4 are all targeted into mitochondria [17], and genetic and biochemical data have shown that they all are related to the splicing of mitochondrial group II introns in Arabidopsis or maize. nMAT1 is essential for the splicing of mitochondrial intron 1 of *nad1*, intron 1 of *nad2*, and intron 2 of *nad4* in Arabidopsis [18]. nMAT2 facilitates the splicing efficiencies of 11 mitochondrial group II introns in Arabidopsis, including introns 2 and 3 of *nad1*; introns 1 and 4 of *nad2*; intron 2 of *nad4*; introns 1, 2, and 3 of *nad5*; intron 2 of *nad7*; and the intron of *rps3* and *cox2* [17,19]. nMAT3 and nMAT4 seem to be related in evolution [20], and they are both found to function during the splicing of mitochondrial *nad1* introns 1, 3, and 4 in Arabidopsis or/and maize [21,22,23].

### 3.2. PPR Proteins

Pentatricopeptide repeat (PPR) proteins typically have tandem arrays of a motif with 35 amino acids [24]. Based on their motif architecture, PPR proteins are classified into classes of P and PLS. P-class PPR proteins always harbor PPR (P) motifs with 35 amino acids, while PLS-class PPR proteins usually contain P, L (longer), and S (shorter) motifs that form tandemly repeated PLS triplets [25]. Based on the conserved C-terminal domains following the tandem arrays of PLS triplets, the PLS-class PPR proteins are classified into subclasses of PLS, E, E+, and DYW. PPR proteins are widely present in land plants and compose a large RNA-binding protein family. Most known PPR proteins are imported into mitochondria and/or chloroplasts and function in multiple steps of RNA metabolism [26,27]. The P-class PPR proteins generally function in diverse aspects of organellar RNA processing, such as RNA splicing, RNA stabilization, and RNA cleavage or translation, while the PLS-class PPR proteins mostly function in RNA editing [28]. Recently, increasing genetic and biochemical data indicate that PPR proteins have critical functions in the splicing of organellar group II introns in land plants. A detailed summary of PPR proteins involved in mitochondrial group II intron splicing in maize and Arabidopsis is listed in Table 1.

Most characterized PPR proteins are needed for the splicing of one or several group II introns in the mitochondria of land plants. For instance, DEK2, EMP16, and PPR18 in maize [29,39,44] and EMB2794, MID1, and OTP43 in Arabidopsis [54,55,59] are specifically involved in the splicing of a single group II intron in mitochondria. DEK41, EMP8, and PPR278 in maize [32,35,47] and BLX, MISF68, and MISF74 in Arabidopsis [53,57] are involved in the splicing of several group II introns in mitochondria. Additionally, two P-class PPR proteins in maize, PPR-SMR1 and SPR2, participate in the splicing of a large proportion of mitochondrial group II introns. PPR-SMR1 has a small MutS-related (SMR) domain at the C-terminus and functions in the splicing of 16 mitochondrial group II introns in maize [48]. SPR2 is a small PPR protein merely harboring four PPR motifs, and it is required for the splicing of 15 mitochondrial group II introns in maize [49].

### 3.3. mTERF Proteins

Similar to PPR proteins, mitochondrial transcription termination factors (mTERFs) are characterized by harboring various numbers of tandem repeats of mTERF motifs, and each mTERF motif contains 30 amino acids forming three α-helices [63]. mTERF proteins are widely present in metazoans, green alga, and plants [63]. In metazoans, the mTERF proteins have been grouped into four subfamilies, mTERF1 to mTERF4, which target mitochondria, bind to nucleic acids, and then regulate DNA replication, transcription, or translation of mitochondrial genes [64,65,66,67,68]. In land plants, there are more members of mTERFs; 31 mTERFs in maize and 35 mTERFs in Arabidopsis have been identified, respectively [69,70]. However, only 14 mTERFs have been well functionally identified in plants; they are all localized in chloroplasts and/or mitochondria and regulate organellar gene expression [71]. The present studies indicate that plant mTERFs can regulate the expression of organellar genes at transcriptional or post-transcriptional levels, including transcription [72], intron splicing [73], and translation [74]. Two plant mTERFs have been shown to be involved in mitochondrial group II intron splicing. The mTERF15 in Arabidopsis is required for the splicing of mitochondrial *nad2* intron 3 [75]. ZmSMK3, an mTERF protein in maize, contains two mTERF motifs and plays an important role in the splicing of the fourth *nad1* intron and the first *nad4* intron in mitochondria [76].

### 3.4. CRM Domain Proteins

CRM domain proteins have a conservative RNA-binding domain denoted chloroplast RNA splicing and ribosome maturation (CRM) domain. The CRM domain is derived from an ancient ribosome-associated protein that has been maintained in eukaryotes only within the genomes of algae and plants [77,78]. In archaea and bacteria, CRM domains exist as a stand-alone protein encoded by single-copy genes, while in plants, they present as a family of proteins containing one to four CRM domains [77]. There are 16 CRM domain proteins in Arabidopsis and 14 in rice, and according to the domain organization, they can be divided into four subfamilies, chloroplast RNA splicing 1 (CRS1) and CRS2 associated factor (CAF), 3, and 4 [77]. Most known CRM domain proteins are involved in RNA splicing in plant chloroplasts or mitochondria. To date, at least 10 CRM domain proteins have been confirmed to promote group II intron splicing in chloroplasts, such as CRS1, CAF1, CRM family member 2 (CFM2), and CFM3 [79]. These CRM domain proteins are required for the splicing of nearly all group II introns in chloroplasts, and group II introns spliced by each CRM domain protein are overlapping but not identical.

The functions of CRM domain proteins in chloroplasts have been well characterized, but their roles in the splicing of mitochondrial introns have rarely been studied. Arabidopsis mitochondrial CAF-like splicing factor 1 (mCSF1) is a member of the CAF subfamily, and it contains two CRM domains. mCSF1 has been demonstrated to be localized exclusively to mitochondria and to be involved in the splicing of 13 group II introns in mitochondria, including the intron of *cox2* and *rps3*; introns 2 and 3 of *nad1*; introns 1, 2, 3, and 4 of *nad2*; introns 1, 2 and 3 of *nad5*; and intron 2 of *nad7* [80]. Zm-mCSF1 is the ortholog of Arabidopsis mCSF1 in maize, and it has two CRM domains and is required for the splicing of six mitochondrial group II introns, including introns 2 and 3 of *nad2*, introns 1 and 2 of *nad5*, intron 3 of *nad7*, and the intron of *ccmFc* [48]. CFM6 to CFM9, members of subfamily 3, harbor one CRM domain and are proposed to localize to mitochondria or nuclei [77]. Genetic and biochemical data imply that Arabidopsis CFM9 targets mitochondria and mediates the splicing of 17 group II introns, including *nad1* introns 1, 2, and 3; *nad2* introns 1, 2, 3, and 4; *nad4* introns 2 and 3; *nad5* introns 1, 2, and 3; *nad7* introns 1, 2 and 4; the *rps3* intron; and the *cox2* intron [81]. Arabidopsis CFM6 was recently characterized to localize to mitochondria and to be specifically involved in the splicing of *nad5* intron 4 [82]. Loss-of-function mutations in these mitochondrial CRM domain proteins generally hinder the assembly and function of mitochondria, and then result in severe retarded growth or defective seed development [48,80,81,82].

### 3.5. DEAD-Box RNA Helicase

RNA helicases are enzymes that catalyze the unwinding of duplex RNA and the rearrangement of ribonucleoprotein complexes [83,84]. Based on their conserved motifs and structures, RNA helicases are divided into six superfamilies, SF1 to SF6 [85]. DEAD-box RNA helicases constitute the largest subfamily of SF2. They are characterized by nine conserved motifs and named after the conservative amino acid sequence of DEAD (Asp-Glu-Ala-Asp) in motif II [86]. Nine conserved motifs of DEAD-box RNA helicases constitute the helicase core that has been reported to be essential for ATP binding, ATP hydrolysis, and RNA binding [87,88,89,90]. DEAD-box RNA helicases exist in prokaryotes and eukaryotes, where they play important functions in RNA processing [91]. About 60 DEAD-box RNA helicase genes have been found in plants [92], but most of them have not been functionally identified. Only two members in Arabidopsis and one member in maize have been characterized to be required for the splicing of mitochondrial group II introns. The putative mitochondrial RNA helicase 2 (PMH2) was characterized to be essential for the splicing of 15 group II introns in Arabidopsis mitochondria, including *nad1* introns 2 and 3; *nad2* introns 1, 2, and 4; *nad4* introns 2 and 3; *nad5* introns 1, 2, and 3; *nad7* introns 1 and 4; *rps* 3 intron; *cox2* intron; and *rpl2* intron [93]. ABA OVERLY SENSITIVE 6 (ABO6) is related to the splicing of 12 mitochondrial group II introns in Arabidopsis, including *nad1* introns 1, 2, 3, and 4; *nad2* introns 3 and 4; *nad4* introns 1, 2, and 3; and *nad5* introns 1, 2, and 3 [94]. Maize ZmRH48 was recently found to be required for the splicing of mitochondrial *nad2* intron 2; *nad5* intron 1; *nad7* introns 1, 2, and 3; and the *ccmFc* intron [95].

### 3.6. Other Proteins

The plant organelle RNA recognition (PORR) domain was previously designated as the ‘domain of unknown function 860′ (DUF860) and was later renamed as the PORR domain because of its RNA-binding ability [96]. PORR proteins constitute a small family in angiosperms, with 15 in Arabidopsis, 15 in maize, and 17 in rice [96]. Nearly all PORR proteins in land plants are postulated to be located in chloroplasts or mitochondria, and two PORR proteins have been determined to function in plant mitochondrial group II intron splicing. WTF9 is required for the splicing of introns in mitochondrial *rpl2* and *ccmFc* in Arabidopsis [97]. OsPORR1 is associated with *nad4* intron 1 splicing in rice mitochondria [98].

The regulator of chromosome condensation 1 (RCC1) proteins typically contain RCC1-like domains. The RCC1-like domain was first identified in RCC1 [99] and has tandemly repeated RCC1 motifs of a conservative domain with about 50 amino acids [100]. RCC1 proteins are predicted to be critical to the regulation of nuclear gene expression. In Arabidopsis and maize, 25 and 31 RCC1 proteins have been identified, respectively, but only two of them, RUG3 and DEK47, have been found to be splicing factors in mitochondria. RUG3 contains seven RCC1 motifs and is essential to the splicing of mitochondrial *nad2* introns 2 and 3 in Arabidopsis [101]. DEK47 also contains seven RCC1 motifs and is involved in the splicing of introns 1, 2, 3, and 4 of the *nad2* transcript in maize mitochondria [102]. As the RNA-binding activity of RCC1 proteins has not been proven, they are predicted to recruit RNA-binding factors or other proteins to the splicing complex [101,103].

Additionally, the organelle zinc finger protein 2 (OZ2) contains two ran-binding protein 2 (RanBP2) zinc finger domains and promotes the splicing of seven group II introns in Arabidopsis mitochondria, including intron 1 of *nad1*; intron 3 of *nad2*; introns 1, 2, and 3 of *nad5*; intron 2 of *nad7*; and the intron of *rps3* [104].

## 4. Roles of Protein Splicing Factors in Mitochondrial Group II Intron Splicing

### 4.1. Multiple Protein Factors Are Needed for the Splicing of a Group II Intron in Land Plant Mitochondria

**Figure 3:**
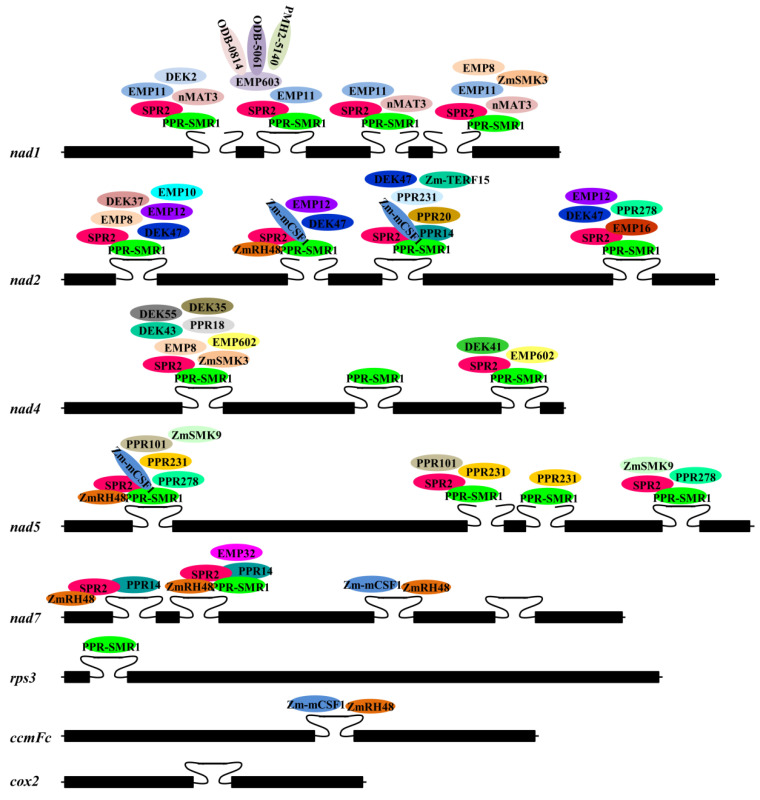
Group II introns and protein factors involved in their splicing in maize mitochondria. The black boxes represent the exons. The closed curves represent the *cis*-spliced introns. The open curves represent the *trans*-spliced introns. The different colored ellipses represent different protein splicing factors. The partially overlapped ellipses represent proteins that have been shown to interact with each other.

**Figure 4:**
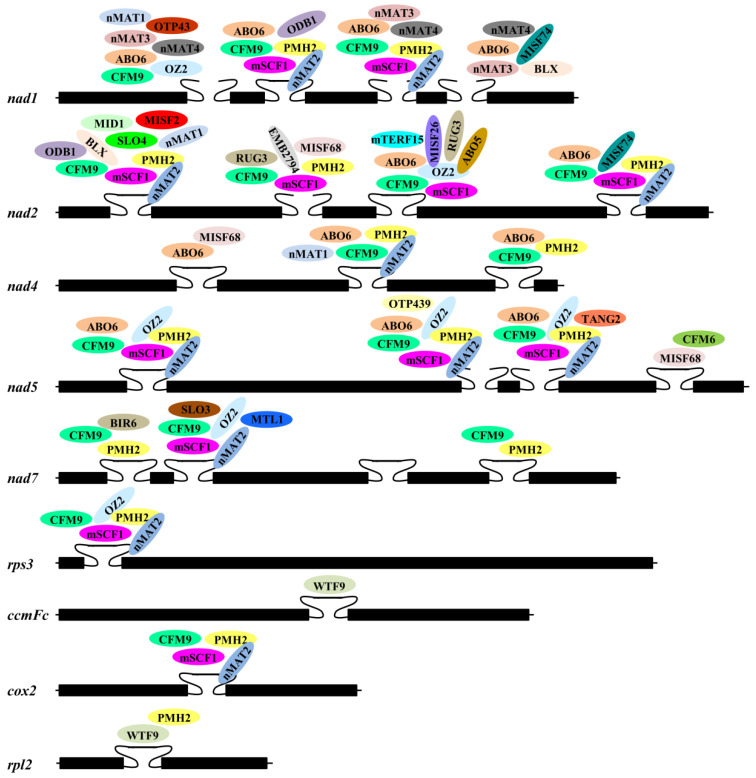
Group II introns and protein factors involved in their splicing in Arabidopsis mitochondria. The black boxes represent the exons. The closed curves represent the *cis*-spliced introns. The open curves represent the *trans*-spliced introns. The different colored ellipses represent different protein splicing factors. The partially overlapped ellipses represent proteins that have been shown to interact with each other.

Accumulating data indicate that the splicing of a mitochondrial group II intron in land plants generally relies on the participation of many protein factors. A detailed summary of proteins required for mitochondrial group II intron splicing in maize and Arabidopsis is provided in Figure 3 and Figure 4, respectively. For instance, the splicing of mitochondrial *nad4* intron 1 in maize requires DEK35, DEK43, DEK55, EMP8, EMP602, PPR18, ZmSMK3, PPR-SMR1, and SPR2 [30,33,34,35,41,44,48,49,76] (Figure 3). In Arabidopsis mitochondria, eight nuclear-encoded proteins from different families, nMAT2, PMH2, ABO6, mCSF1, CFM9, SLO4, MID1, and ODB1, are involved in *nad1* intron 2 splicing [17,55,62,80,81,93,94,105] (Figure 4).

In land plant mitochondria, multiple protein factors being required for the splicing of one group II intron hints the potential involvement of a splicing complex. Indeed, increasing studies indicate that large ribonucleoprotein complexes associated with the splicing of group II introns may exist in the mitochondria of land plants. Both nMAT2 and PMH2 are required for the splicing of 10 out of 23 group II introns in Arabidopsis mitochondria [19] (Figure 4). Analysis of pull-down experiments and native mitochondrial extracts showed the interaction between nMAT2 and PMH2 and the presence of nMAT2, PMH2, and their intron RNA targets in a large ribonucleoprotein complex. OZ2 functions in the splicing of seven group II introns in Arabidopsis mitochondria [104]. Three mitochondrial splicing factors, ABO5 [51], MISF26 [57] and PMH2 [93], share target introns with OZ2, and physical interactions between OZ2 and these three proteins were observed in yeast two-hybrid and bimolecular fluorescence complementation assays [104]. EMP603, a PPR protein, is specifically involved in mitochondrial *nad1* intron 2 splicing in maize and interacts with DEAD-box RNA helicase PMH2-5140, the RAD52-like proteins ODB1-0814 and ODB1-5061, and Zm-mCSF1 *in vitro* and *in vivo* [42].

PPR-SMR1 and SPR2 in maize are involved in the splicing of 16 and 15 group II introns in mitochondria, respectively, and they are both essential for the splicing of 13 out of 22 group II introns in mitochondria [48,49] (Figure 3). PPR14, Zm-mCSF1, and EMP16 are specifically involved in the splicing of one or several introns of these 13 introns [39,43,48]. Bimolecular fluorescence complementation and pull-down experiments revealed that PPR-SMR1, PPR14, and Zm-mCSF1 directly interact with one another [43,48], SPR2 directly interacts with PPR-SMR1, Zm-mCSF1, PPR14, and EMP16 [49]. Meanwhile, the luciferase complementation imaging assay indicated that the interaction of PPR-SMR1 with EMP16 is mediated by the bridging of SPR2 [49]. Moreover, ZmRH48 in maize was recently reported to be involved in the splicing of three out of 13 mitochondrial group II introns spliced by both PPR-SMR1 and SPR2 (Figure 3), and direct interactions between ZmRH48 and PPR-SMR1, SPR2, Zm-mCSF1 were confirmed through pull-down and bimolecular fluorescence complementation experiments [76].

These data imply that the splicing of group II introns in plant mitochondria is also performed by a spliceosomal complex. Splicing factors act as components of a spliceosomal complex in the mitochondria of land plants, in which some splicing factors, such as SPR2/PPR-SMR1 in maize and nMAT2/PMH2 in Arabidopsis, are proposed to serve as the core components of the spliceosomal complex and exert intron splicing through dynamic interactions with other intron-specific splicing factors in plant mitochondria.

### 4.2. Roles of Protein Splicing Factors in the Splicing of Mitochondrial Group II Introns in Land Plants

In group II introns, the formation of exact and conserved ribozyme-like tertiary structures is required for their removal from pre-RNAs. Compared with bona fide group II introns in bacteria, group II introns in plant organelles have degenerated and lost some essential fragments required for the formation of the active tertiary structures. Factors of splicing machinery in land plant organelles are thought to aid group II introns in folding into the correct structure suitable for splicing. In chloroplasts, several splicing factors have been shown to promote or maintain a proper intron structure by direct intron binding, such as CRS1, PPR4, and PpPPR_66 [79].

During the splicing process, the exact roles of splicing factors in plant mitochondrial group II intron RNA folding or sequence recognition have not been established. Arabidopsis nMAT1 may function in the release of 5′ exons from its target introns [20]. Two DEAD/DExH-box RNA helicases in Arabidopsis, PMH2 and ABO6, are predicted to function as RNA chaperones required for resolving stable inactive secondary structures within introns and promoting introns to fold correctly in mitochondria [93,94]. ODB1, a RAD52-like protein, is thought to facilitate the splicing of intron 2 in *nad1* and intron 1 in *nad2* by stabilizing the correctly folded structures of DV and DVI in the two introns either through directly binding to the introns or interacting with other components of the spliceosomal complex [105]. In Arabidopsis mitochondria, nMAT2, PMH2, and their intron RNA targets are proposed to be present in a large ribonucleoprotein particle [19], implying that nMAT2 and PMH2 promote the splicing of their target introns by directly binding to intron RNAs or interacting with other splicing factors in a spliceosomal complex.

Among the known mitochondrial splicing factors, PPR proteins make up the largest group. As members of the RNA-binding protein family, some PPR proteins facilitate RNA editing in plant organelles by specifically binding to their target RNAs through a one repeat–one nucleotide mechanism [106,107,108]. Additionally, the PPR proteins EMB2654, OTP51, PBF2, PTSF1, PpPPR_66, PPR4, and THA8 functioning in group II intron splicing in chloroplasts have been shown to directly bind to their target introns *in vitro* [79]. In plant mitochondria, more than thirty PPR proteins have been found to function in mitochondrial group II intron splicing in land plants to date (Table 1), and several of them have been identified to interact with various mitochondrial splicing factors *in vivo* and *in vitro* [48,49]. Accordingly, it is hypothesized that PPR proteins function predominantly by recognizing and binding to specific RNA sequences within their target introns, binding with other splicing factors to promote or maintain the intron RNA folding into a correct active conformation, and then initiating splicing.

These data suggest that splicing factors from diverse families may function in the splicing of plant mitochondrial group II introns by recognizing target RNA sequences or interacting with other splicing factors to form or maintain the active spliceosomal complex. However, further biochemical studies are necessary to confirm their exact functions in the splicing of group II introns in plant mitochondria.

## 5. Conclusions and Prospects

Due to the loss or degeneration of sequences encoding IEPs, the splicing of plant mitochondrial group II introns is promoted by protein cofactors. Indeed, some splicing factors in plant mitochondria have been reported (Figure 3 and Figure 4), but only a few of them have been confirmed to function by binding to their target intron RNAs or interacting with other splicing factors [19,20,42,48,49,76,104,105]; the precise roles of most splicing factors in the splicing of plant mitochondrial group II introns are not yet understood. Group II introns are considered to be the ancestor of spliceosomal introns in nuclei; thus, it is proposed that group II intron splicing *in vivo* is promoted by a spliceosome. Multiple protein factors being involved in the splicing of one group II intron in plant mitochondria hints the potential involvement of a splicing complex (Figure 3 and Figure 4); however, it remains largely unclear what components are present in a splicing complex and whether they form a larger complex in the same manner as a nuclear spliceosome. Most known splicing-related proteins are members of the PPR protein family, and more than twenty PPR proteins in maize have been identified to be involved in the splicing of group II introns in mitochondria (Table 1). The direct binding of PPR proteins to their target RNAs has been found in RNA editing and in the splicing of chloroplast group II introns [79,106,107,108], while little is known about the interaction between PPR proteins and their target introns in plant mitochondria. In addition, some splicing factors have been characterized for most introns in mitochondrial genes, but very few have been reported in several mitochondrial genes, such as *cox2*, *ccmFc,* and *rps3* in maize mitochondria and *rpl2* and *ccmFc* in Arabidopsis mitochondria (Figure 3 and Figure 4). Thus, more biochemical analysis techniques, such as gel shift and co-immunoprecipitation, are needed to confirm the presence of a spliceosome in plant mitochondria, the binding of splicing factors to specific RNA sequences in their target introns, or the interaction of splicing factors with other components of the spliceosome. More genetic studies are needed to identify more splicing factors in plant mitochondria, especially the splicing factors associated with the splicing of introns of *cox2*, *ccmFc,* and *rps3* in maize mitochondria and *rpl2* and *ccmFc* in Arabidopsis mitochondria.

## Figures and Tables

**Table 1:** List of mitochondrion-targeted PPR proteins required for the splicing of mitochondrial group II introns in maize and Arabidopsis.

Species	Protein Name	PPR Class	Target Introns	References
Maize	DEK2	P	*nad1* intron 1	[29]
	DEK35	P	*nad4* intron 1	[30]
	DEK37	P	*nad2* intron 1	[31]
	DEK41/DEK43	P	*nad4* introns 1 and 3	[32,33]
	DEK55	PLS	*nad1* introns 1 and 4; *nad4* intron 1	[34]
	EMP8	P	*nad1* intron 4; *nad2* intron 1; *nad4* intron 1	[35]
	EMP10	P	*nad2* intron 1	[36]
	EMP11	P	*nad1* introns 1, 2, 3 and 4	[37]
	EMP12	P	*nad2* introns 1, 2 and 4	[38]
	EMP16	P	*nad2* intron 4	[39]
	EMP32	P	*nad7* intron 2	[40]
	EMP602	P	*nad4* introns 1 and 3	[41]
	EMP603	P	*nad1* intron 2	[42]
	PPR14	P	*nad2* intron 3; *nad7* introns 1 and 2	[43]
	PPR18	P	*nad4* intron 1	[44]
	PPR20	P	*nad2* intron 3	[45]
	PPR101	P	*nad5* introns 1 and 2	[46]
	PPR231	P	*nad5* introns 1, 2 and 3; *nad2* intron 3	[46]
	PPR278	P	*nad2* intron 4; *nad5* introns 1 and 4	[47]
	PPR-SMR1	P	*nad1* introns 1, 2, 3 and 4; *nad2* introns 1, 2, 3 and 4; *nad4* introns 1, 2 and 3; *nad5* introns 1, 3 and 4; *nad7* intron 2; *rps3* intron	[48]
	SPR2	P	*nad1* introns 1, 2, 3 and 4; *nad2* introns 1, 2, 3 and 4; *nad4* introns 1 and 3; *nad5* introns 1, 2 and 4; *nad7* introns 1 and 2	[49]
	ZmSMK9	P	*nad5* introns 1 and 4	[50]
Arabidopsis	ABO5	P	*nad2* intron 3	[51]
	BIR6	P	*nad7* intron 1	[52]
	BLX	PLS	*nad1* intron 4; *nad2* intron 1	[53]
	EMB2794	P	*nad2* intron 2	[54]
	MID1	P	*nad2* intron 1	[55]
	MISF2	P	*nad2* intron 1	[56]
	MISF26	P	*nad2* intron 3	[57]
	MISF68	P	*nad2* intron 2; *nad4* intron 1; *nad5* intron 4	[57]
	MISF74	P	*nad1* intron 4; *nad2* intron 4	[57]
	MTL1	P	*nad7* intron 2	[58]
	OTP43	P	*nad1* intron 1	[59]
	OTP439	P	*nad5* intron 2	[60]
	TANG2	P	*nad5* intron 3	[60]
	SLO3	P	*nad7* intron 2	[61]
	SLO4	P	*nad2* intron 1	[62]

## Data Availability

Not applicable.
